# The Diagnosis of Pancreatic Disease

**Published:** 1907-09-21

**Authors:** 


### THE DIAGNOSIS OF PANCREATIC DISEASE.

### CAMMIDGE'S REACTION.

The difficulty of diagnosing the presence of disease of the pancreas is a matter of common experience, and such disease often escapes recognition during the life of the patient. The principal clinical signs and symptoms on which the diagnosis is made are (1) the presence of undigested fat in the stools, (2) an epigastric tumour, and (3) glycosuria. The simultaneous presence of all three is highly suggestive, although not absolutely pathognomonic, of pancreatic disease; any one of them alone is quite useless as evidence. For instance, undigested fat may be found in obstruction of the common bile-duct, due to other causes than pancreatic disease, or may be altogether absent when the pancreas is extensively affected; and where this is also the case there may be no glycosuria. Further, there may be advanced disease of the organ without a palpable epigastric tumour; and even when one is present it is not often possible to attribute it definitely to the pancreas. When one remembers the complicated anatomy of the upper half of the abdomen it will be readily understood that such a tumour may have its origin equally well in the gall-bladder, pylorus, liver, or even kidney; and the writer remembers a case of epigastric tumour which was diagnosed as being of pancreatic origin, but which was found on the operating-table to be caused by a mesenteric cyst. If the difficulties which face the surgeon in diagnosing the presence of disease of the pancreas are so great, it is easy to understand that the difficulty of discovering the nature of the disease is immeasurably greater. For the simplification of these difficulties the profession at large is indebted to Dr. P. J. Cammidge, who has invented a reaction which has been named after him, "Cammidge's Pancreatic Reaction." His line of argument is as follows: * "Just as in hepatic disease the constituents of the bile are known to get into the blood and appear in the urine, so in pancreatic disease the constituents of the pancreatic juice may get into the blood and appear in the urine. Now if pancreatic juice were circulating in the blood, one of its possible effects would be the splitting up of the body fat into fatty acids and glycerine. Such a splitting up does occur in acute pancreatitis and is manifested in the phenomenon of fat necrosis. But in the areas of fat necrosis free fatty acids are found; therefore the glycerine must go somewhere. Can it be found in the urine?" Dr. Cammidge asserts that this is the case, and the whole object of his process is to demonstrate this glycerine or glycerine derivative in the urine. He first explained the technique of his method in 1904. Two reactions were then described. In the first, which he calls "A," ten cubic centimetres of the filtered urine are boiled for ten minutes with one cubic centimetre of strong hydrochloric acid. To five cubic centimetres of this mixture is added an equal volume of distilled water. This is allowed to cool, and the excess of acid is neutralised with four grammes of lead carbonate. It is now filtered, and to the filtrate are added two grammes of powdered sodium acetate and three-quarters of a gramme of phenylhydrazin hydrochlorate, and the resulting mixture is boiled. After cooling, a yellow deposit separates out, which consists microscopically of sheaves of crystals. It is, of course, necessary to remove previously any sugar which may be present at the start, and this may be done by fermentation. Albumen, too, if present, hinders the reaction, and should be got rid of. This reaction alone he found to be not absolutely trustworthy, since a positive result was obtained in diseases where active tissue change was progressing—*e.g.*, pneumonia. But he subsequently discovered a means of differentiating between crystals of pancreatic and those of non-pancreatic origin in reaction "A"; since if the urine is treated with a solution of perchloride of mercury, the formation of crystals of pancreatic origin is inhibited. Hence reaction "B." Twenty cubic centimetres of the filtered urine are mixed with ten cubic centimetres of a saturated solution of perchloride of mercury. This is filtered and the filtrate boiled with one cubic centimetre of strong hydrochloric acid. After cooling, it is neutralised with four grammes of lead carbonate, and the rest of the reaction is carried out as in reaction "A." According to Dr. Cammidge, the nature of the disease affecting the pancreas may be inferred according to the length of time necessary for complete solution of the crystals in a 33 per cent. solution of sulphuric acid. This is obtained by observing their behaviour under the microscope. In acute pancreatitis solution occurs in from thirty to forty-five seconds; in chronic pancreatitis it takes from half a minute to two minutes, and in malignant disease of the pancreas from three to five minutes. Further than this, he summarises his results as follows: (1) If no crystals are found by either the "A" or "B" method the pancreas is not diseased. (2) If crystals are obtained by the "A" method, but not by the "B," the pancreas is inflamed, and surgical interference is indicated. The actual state of inflammation (whether acute or chronic) can be determined by the time required for the solution of the crystals in sulphuric acid, as explained above. (3) If crystals are formed by both methods there may be (*a*) malignant disease of the pancreas, when the crystals will dissolve in from three to five minutes; or (*b*) some disease not connected with the pancreas, when the crystals will dissolve in about one minute. More recently (1906) Dr. Cammidge has described an improved method of performing the reaction. It differs from the original chiefly in the fact that the large amount of glycuronic acid present in the urine in most cases of pancreatic disease is removed by treatment with basic lead acetate, thus allowing a sugar which appears to be constantly obtainable in inflammatory conditions to be more readily recognised. Several workers have recently tested a number of cases with this reaction, but their results do not appear to be uniform, and the method has been subjected to criticism; but in view of the statement of Mr. Mayo Robinson, "The test is certainly of great help in diagnosis," it assuredly deserves an extended trial.

* Dr. Taylor, *Lancet*, June 30, 1906.

