# The Diagnosis of Pancreatic Disease

**Published:** 1907-10-05

**Authors:** 


A Correction.—We regret that in the article on "The Diagnosis of Pancreatic Disease" in The Hospital of September 21, 1907, page 660, in which mention was made of Mr. Mayo Robson's work, the name of this authority was inadvertently misquoted.

